# Primate embryogenesis predicts the hallmarks of human naïve pluripotency

**DOI:** 10.1242/dev.145177

**Published:** 2017-01-15

**Authors:** Thorsten Boroviak, Jennifer Nichols

**Affiliations:** 1 Wellcome Trust-Medical Research Council Cambridge Stem Cell Institute, University of Cambridge, Tennis Court Road, Cambridge CB2 1QR, UK; 2 Department of Physiology, Development and Neuroscience, University of Cambridge, Downing Street, Cambridge CB2 4BG, UK

**Keywords:** Amnion, Epiblast, Extraembryonic, Naïve pluripotency, Postimplantation, Primate

## Abstract

Naïve pluripotent mouse embryonic stem cells (ESCs) resemble the preimplantation epiblast and efficiently contribute to chimaeras. Primate ESCs correspond to the postimplantation embryo and fail to resume development in chimaeric assays. Recent data suggest that human ESCs can be ‘reset’ to an earlier developmental stage, but their functional capacity remains ill defined. Here, we discuss how the naïve state is inherently linked to preimplantation epiblast identity in the embryo. We hypothesise that distinctive features of primate development provide stringent criteria to evaluate naïve pluripotency in human and other primate cells. Based on our hypothesis, we define 12 key hallmarks of naïve pluripotency, five of which are specific to primates. These hallmarks may serve as a functional framework to assess human naïve ESCs.

## Abstract (precis)

**Summary:** This Hypothesis article highlights several fundamental differences between rodent and primate early development and exploits these to predict key hallmarks of naïve pluripotency in primates.

## Introduction

Embryonic stem cells (ESCs) have been derived from preimplantation embryos of a variety of non-rodent mammals, including rabbit (Graves and Moreadith, 1993), cow (Gjørret and Maddox-Hyttel, 2005), pig (Notarianni et al., 1991), sheep (Notarianni et al., 1991), marmoset monkey (Sasaki et al., 2005; Thomson et al., 1996), rhesus monkey (Thomson et al., 1995) and human (Ludwig et al., 2006; Thomson et al., 1998). However, in contrast to mouse and rat ESCs, none of the cell lines from non-rodent species has convincingly demonstrated contribution to chimaeras when injected into a host embryo. Conventional human ESCs have a dramatically different transcriptome and methylome compared with the inner cell mass (ICM) of the human blastocyst from which they derive (Guo et al., 2014; Yan et al., 2013). This suggests that the conditions in which the cells are cultured fail to capture the transient developmental programme of the embryo. Instead, human and non-human primate ESCs share distinctive features with cells derived from the mouse postimplantation epiblast, which has led to the proposition that they represent a later stage of development (Brons et al., 2007; Nichols and Smith, 2009; Tesar et al., 2007). These findings have sparked efforts to reset conventional primate, and in particular human, ESCs to an earlier developmental state, more akin to mouse ESCs. These approaches were initially dependent upon overexpression of potent pluripotency factors (Buecker et al., 2010; Hanna et al., 2010; Li et al., 2009; Wang et al., 2011), but recently several culture conditions were reported in which it is possible to convert conventional human ESCs from ʻprimed' (postimplantation) to ʻnaïve' (preimplantation) pluripotency in the absence of continuous transgene expression (Chan et al., 2013; Chen et al., 2015a,b; Duggal et al., 2015; Gafni et al., 2013; Takashima et al., 2014; Theunissen et al., 2014; Valamehr et al., 2014; Ware et al., 2014). Since ethical considerations prohibit the functional evaluation of these putatively naïve pluripotent human ESCs in germline chimaera assays, stringent criteria are needed to define naïve pluripotency in human and other primates.

In this Hypothesis article, we advocate that preimplantation epiblast identity is imperative for the naïve state in human and non-human primates. We discuss the fundamental differences between primate and rodent development and hypothesise that these differences might provide stringent criteria to evaluate naïve pluripotency in human and other primate cells. Based on this hypothesis, we extract 12 hallmarks of naïve pluripotency from early histological studies and recent discoveries in primate embryology. Seven of these equally apply to mouse ESCs; the remaining five reflect the primate-specific adaptations of early development. Our hypothesis provides a testable framework to assess naïve pluripotency in primates – a timely requirement in the light of recent achievements in resetting human ESCs.

## Capturing pluripotent states from the embryo

Mammalian embryos establish an unrestricted state of embryonic potential in the epiblast prior to implantation. After fertilisation, the unicellular zygote undergoes several rounds of cleavage divisions, resulting in a progressively greater number of increasingly smaller cells. These cells are called blastomeres and subsequently go through compaction. During this process, the outer cells establish apical-basal polarity and are directed towards the trophoblast lineage, a prerequisite for blastocyst formation. Interior cells become ICM and gradually diverge into pluripotent epiblast and extraembryonic hypoblast (also called primitive endoderm) (Chazaud et al., 2006; Frankenberg et al., 2011; Plusa et al., 2008; Ralston and Rossant, 2008; Rossant and Tam, 2009; Schrode et al., 2014; Strumpf et al., 2005). At the mid-to-late blastocyst stage, cleavage ceases (Aiken et al., 2004) as cells gain the capacity to replenish cytosol and organelles before division and become autopoietic (ʻself-creating'). The ICM lineages segregate irreversibly (Grabarek et al., 2012) and the founding cell population of the foetus is established in the preimplantation epiblast.

In mouse, this stage of development can be captured in the form of ESCs (Brook and Gardner, 1997; Evans and Kaufman, 1981; Martin, 1981). ESCs cultured with mitogen-activated protein kinase kinase (Mek) and Gsk3β inhibition plus leukaemia inhibitory factor (2i/LIF) (Ying et al., 2008) correspond to the preimplantation epiblast in terms of gene expression (Boroviak et al., 2014) and functionally contribute to chimaeras upon injection into a host blastocyst (Bradley et al., 1984; Ying et al., 2008). The unrestricted potential of preimplantation epiblast and ESCs to give rise robustly to all somatic lineages and the germline has been termed ʻnaïve' pluripotency (Nichols and Smith, 2009). By contrast, cell lines derived from the mouse postimplantation epiblast are called epiblast stem cells (EpiSCs). Although EpiSCs express several pluripotency factors and differentiate into the three germ layers *in vitro* as well as in teratoma assays, they have lost their ability to re-enter embryonic preimplantation development consistently in blastocyst chimaera assays (Brons et al., 2007; Tesar et al., 2007). However, they do contribute to somatic lineages when introduced into the postimplantation embryo *in vitro* (Huang et al., 2012) and express early markers of lineage specification (Brons et al., 2007; Tesar et al., 2007). EpiSCs share features, including gene expression, with anterior primitive streak cells of the late gastrula, a cell population heterogeneously ʻprimed' for successive lineage commitment (Kojima et al., 2014). This renders EpiSCs predisposed to differentiate into germ layer derivatives to a variable degree (Bernemann et al., 2011; Kojima et al., 2014). Therefore, this stage of pluripotency is referred to as ʻprimed' (Nichols and Smith, 2009).

Primate ESCs in conventional culture conditions containing knockout serum replacement (KSR) and basic fibroblast growth factor (bFGF; also known as FGF2) have consistently failed to produce chimaeras (Okano et al., 2012) and share distinctive features with primed mouse EpiSCs, despite their blastocyst origin (Brons et al., 2007; Tesar et al., 2007). Conventional primate ESCs rely on FGF and activin/Nodal signalling for self-renewal and exhibit a flat colony morphology, low clonogenicity, repressive epigenetic marks, and differentiation bias (Bernemann et al., 2011; Brons et al., 2007; Han et al., 2010; Nichols and Smith, 2009; Tesar et al., 2007). Recent transcriptome analysis of primate pre- and postimplantation embryos revealed that human and monkey ESCs show highest similarity to the late postimplantation epiblast (Nakamura et al., 2016). This confirms the proposition that primate ESCs in conventional culture represent a later developmental state than mouse ESCs (Brons et al., 2007; Nichols and Smith, 2009; Rossant, 2008; Tesar et al., 2007).

In rodents, primed cells can be reverted to a naïve state from EpiSCs (Festuccia et al., 2012; Guo et al., 2009; Martello et al., 2013; Yang et al., 2010) and from the *in vivo* postimplantation epiblast (Bao et al., 2009). A recent flurry of reports described the derivation of so-called naïve pluripotent human ESCs (Chan et al., 2013; Chen et al., 2015a,b; Duggal et al., 2015; Gafni et al., 2013; Takashima et al., 2014; Theunissen et al., 2014; Valamehr et al., 2014; Ware et al., 2014; reviewed by Ávila-González et al., 2016). All of these conditions are modifications of the 2i/LIF culture regime developed for efficient mouse ESC derivation and culture. The majority contain additional cytokines, such as activin A or bFGF and generally require feeder cells. Transcriptome comparison of naïve human ESCs with early embryos suggests that 5i/L/FA (2i/LIF plus inhibitors of BRAF, ROCK and SRC plus activin A and FGF) cells (Theunissen et al., 2014) and t2iL+Gö (2i/LIF with lower, titrated levels of Gsk3β inhibitor plus aPKC inhibitor) reset cells (Takashima et al., 2014) exhibit distinct features of *in vivo* preimplantation development (Huang et al., 2014; Pastor et al., 2016). Chimaeric foetuses have been generated with non-human primate ESCs (Chen et al., 2015b), but low chimaerism and a lack of lineage marker analysis after morula injection prevent definitive conclusions at present. Human ESCs cannot be tested for their full developmental potential to make germline chimaeras for ethical reasons. Analysis of mid-gestation chimaeras for contribution from human ESCs has been met with inconsistent success, marking this controversial technique as an unreliable readout for determining human pluripotency (Gafni et al., 2013; Theunissen et al., 2016). This further highlights the need for alternative functional assays to discriminate between human primed and naïve pluripotent states. We hypothesise that such distinguishing features can be gleaned from early primate development.

## Distinctive features of early primate development

**Fig. 1.**
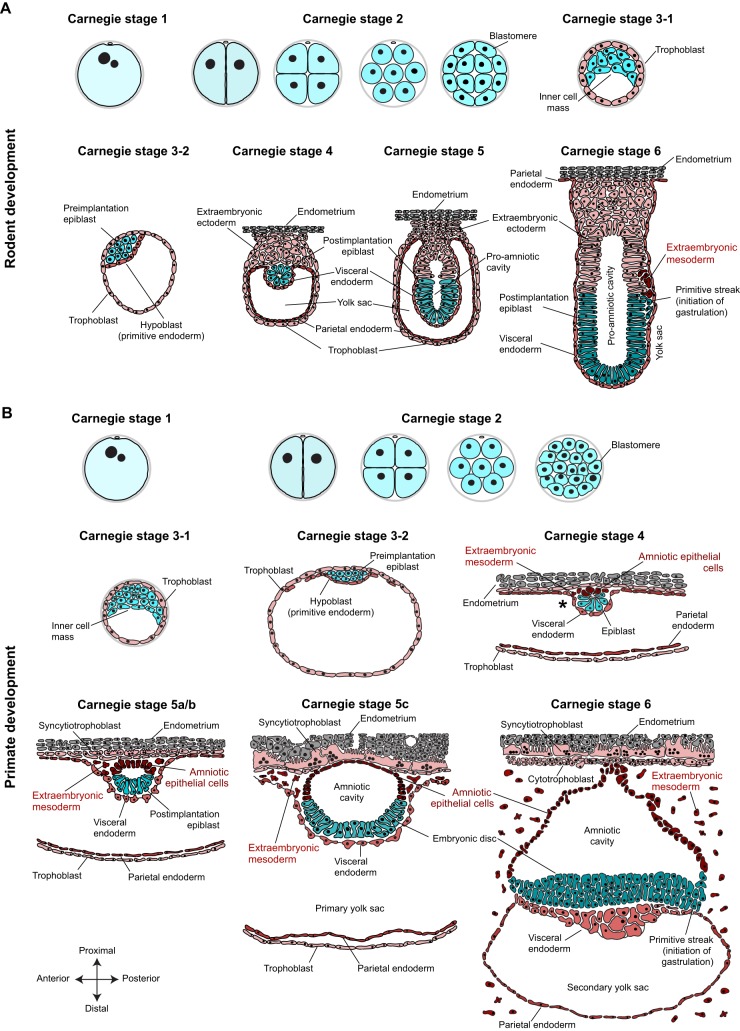
**Schematic overview of rodent and primate development from fertilisation to gastrulation.** Developmental time of rodent development (A) and primate development (B) is given in Carnegie stages to facilitate comparison between species. Embryonic lineages are represented in blue shades, extraembryonic lineages in red shades. Cartoons for primate development were drawn based on histological sections of common marmoset (Moore et al., 1985), rhesus macaque (Enders and King, 1988; Enders et al., 1986) and early human stages of the Carnegie collection (Hertig and Rock, 1941, 1946, 1949; Rock and Hertig, 1948). Note that extraembryonic mesoderm specification from visceral endoderm is exclusively based on electron micrographs of early rhesus macaque implantation stages.

Embryogenesis in primates is protracted compared with rodents. Several differences in developmental timing emerge directly after fertilisation: the pluripotency factor *POU5F1* (*OCT4*) is barely expressed in human embryos until the 8-cell stage, whereas mouse *Pou5f1* transcripts are detected in the zygote and are initially downregulated, then upregulated at the 8-cell stage (Blakeley et al., 2015; Palmieri et al., 1994). Moreover, human embryos activate their genome at the 4- to 8-cell stage (Braude et al., 1988; Vassena et al., 2011; Yan et al., 2013), rather than at the 2-cell stage as in mouse (Flach et al., 1982). Both rodent and primate embryos undergo several rounds of cleavage divisions (Fig. 1A,B, Carnegie stage 2), but compaction occurs slightly later in primates between the 16-cell and the 32-cell stage, as compared with the 16-cell stage in mouse. The first two lineage decisions, however, are conserved between rodents and primates: outer blastomeres form intercellular connections and establish apical-basal polarity, which pre-empts the first lineage decision between ICM and trophoblast (Fig. 1A,B, Carnegie stage 3); the blastocyst expands and subsequently initiates the second lineage decision, whereby ICM cells segregate into pluripotent epiblast and extraembryonic hypoblast by Carnegie stage 3-2 (Fig. 1A,B). Rodent and primate extraembryonic tissues exhibit apical-basal polarity, with the trophoblast facing outwards and hypoblast towards the blastocoel (Enders and Schlafke, 1981; Nadijcka and Hillman, 1974). The cells of the preimplantation epiblast are apolar and remain sandwiched between the basal sides of trophoblast and hypoblast (Enders et al., 1986; Nadijcka and Hillman, 1974). Consequently, both rodent and primate late blastocysts set aside the founding population of the ‘embryo proper’ and specify two extraembryonic lineages for successful attachment to the uterine wall.

Primate development radically diverges from the rodent paradigm during implantation. In mouse, the embryo attaches to the deciduum and epiblast cells arrange themselves radially into a rosette-like structure between trophoblast and hypoblast (Fig. 1A, Carnegie stage 4). Epiblast cells establish junctions at the newly formed apex and concentrate their organelles towards the centre. This transformation requires basal membrane-stimulated integrin signalling and results in the formation of a central cavity (Bedzhov and Zernicka-Goetz, 2014). Trophoblast cells at the proximal end of the implanting embryo expand to form extraembryonic ectoderm and the ectoplacental cone (Fig. 1A, Carnegie stages 4 and 5). The extraembryonic ectoderm also undergoes polarisation and forms a cup-shaped layer of epithelial cells proximal to the epiblast. At the same time, the hypoblast diversifies and expands to form parietal and visceral endoderm. Parietal endoderm migrates along the inner side of the trophoblast. Visceral endoderm overlies both epiblast and extraembryonic ectoderm, predominantly forming the endoderm of the visceral yolk sac (Arnold and Robertson, 2009; Tam and Loebel, 2007), but also contributing to definitive endoderm (Kwon et al., 2008). Although this part of the developmental programme is shared between rodent and primate, there is a clear and crucial exception: the primate embryo establishes two additional extraembryonic lineages at this stage – the amniotic epithelial cells and the extraembryonic mesoderm (Fig. 1B, highlighted in red).

**Fig. 2.**
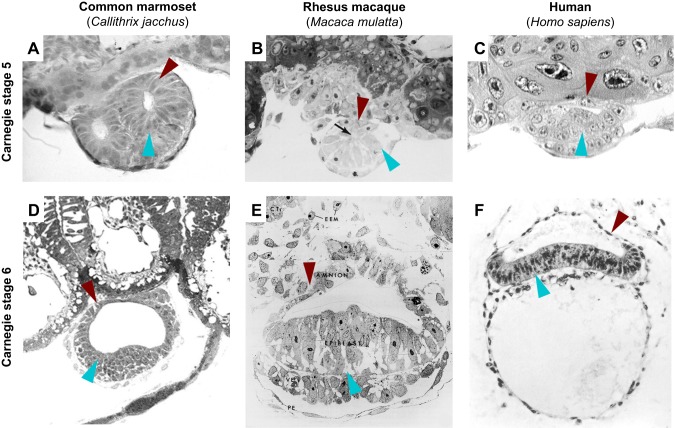
**Images of early primate implantation stages.** Carnegie stages 5 and 6 are shown from (A,D) common marmoset (Enders and Lopata, 1999), (B,E) rhesus macaque (Enders et al., 1986) and (C,F) human stages of the Carnegie collection (Hertig and Rock, 1941; O'Rahilly and Muller, 1987). Blue arrowheads indicate postimplantation epiblast (A-C) or embryonic disc (D-F), red arrowheads indicate amniotic epithelial cells. Images reproduced with permission from John Wiley and Sons (A,B,D,E) and the Carnegie Institution of Washington (C,F).

Primates segregate amniotic epithelial cells directly from the peri-implantation epiblast. During implantation, the primate epiblast forms a rosette-like structure, similar to mouse, with epiblast cells underlying the trophoblast sharing desmosomal junctions with trophoblast cells (Enders et al., 1986). In addition, primate epiblast cells adjacent to the visceral endoderm increase in size and displace the centre of the rosette (Fig. 1B, Carnegie stage 4). Lumen formation in the centre of the implanting rosette gives rise to the amniotic cavity. These rearrangements yield two morphologically distinctive cell types: amniotic epithelial cells, which are the precursors of the amniotic sac, on the cytotrophoblast side; and postimplantation epiblast cells, destined to form the embryonic disc, which reside adjacent to visceral endoderm (Fig. 1B, Carnegie stages 5 to 6). The amnion is a smooth epithelium consisting of low cuboidal cells linked by apical junctional complexes. It is contiguous with the taller, columnar epiblast, reflecting their common origin. Recent progress in the culture of human embryos to early postimplantation stages *in vitro* has allowed the direct observation of amniotic cavity formation (Deglincerti et al., 2016; Shahbazi et al., 2016). Human epiblast cells acquire apical-basal polarity, undergo lumen formation and establish columnar and squamous POU5F1-positive populations, representative of embryonic disc and prospective amniotic epithelium, respectively (Deglincerti et al., 2016; Shahbazi et al., 2016). This direct mode of amnion formation from the preimplantation epiblast before gastrulation is described in marmoset, rhesus macaque and human (Fig. 2), suggesting a conserved feature of primate development. A recent report showing that primate germ cells are specified from amniotic epithelial cells further underlines the major importance of this lineage decision (Sasaki et al., 2016). In mouse, amnion formation is initiated later, at the onset of gastrulation, when extraembryonic mesoderm is specified from the posterior epiblast (Fig. 1A, Carnegie stage 6). This leads to formation of the amniochorionic fold [formerly called the ʻposterior amniotic fold' (Kaufman, 1992)], which gives rise to both amnion and chorion (described by Pereira et al., 2011).

The second fundamental difference between rodent and primate development is extraembryonic mesoderm specification (Fig. 1B, Carnegie stage 4). In rodents, gastrulation initiates in the primitive streak, which is induced at the proximal posterior extremity of the postimplantation epiblast at Carnegie stage 6 (Fig. 1A). Distinct mesodermal cell lineages become allocated according to the time and site of ingression through the streak (Arnold and Robertson, 2009; Lawson, 1999). The earliest population of mouse epiblast cells to undergo epithelial-to-mesenchymal transition and migrate through the streak gives rise to extraembryonic mesoderm, including the mesodermal layer of the chorion, visceral yolk sac mesoderm and blood islands (Arnold and Robertson, 2009). Thus, in rodents, extraembryonic mesoderm formation occurs during gastrulation. By contrast, primates specify extraembryonic mesoderm at implantation, long before gastrulation (Fig. 1B, Carnegie stage 4). Primate visceral endoderm derivatives invade the space between visceral endoderm and cytotrophoblast (Fig. 1B, Carnegie stage 4, asterisk). These subendodermal cells appear in ultrastructure similar to endoderm, but have lost apical junctional complexes and microvilli, and differentiate into extraembryonic mesoderm (Enders and King, 1988). As development progresses, extraembryonic tissues undergo rapid proliferation and displace the embryo away from the cytotrophoblast. Extraembryonic mesoderm cells are stellate in appearance and produce copious extracellular matrix (Enders and King, 1988). The embryonic disc is connected to the developing placenta via a stalk of amnion termed the amniotic diverticulum (Enders et al., 1986). Epiblast cells preferentially divide at the apical surface (Enders et al., 1986), reminiscent of interkinetic nuclear migration in neuroectoderm. The primitive streak is initiated posteriorly at the margin of the embryonic disc at Carnegie stage 6, when epiblast cells start to invade the space towards visceral endoderm. These embryonic mesodermal cells are of primitive and undifferentiated appearance, in contrast to their extraembryonic counterparts, which are characterised by high motility and extracellular matrix production (Enders and King, 1988).

In summary, primates specify two additional extraembryonic lineages before gastrulation, with amniotic epithelial cells directly derived from the peri-implantation epiblast.

## Naïve ESCs are functionally equivalent to the preimplantation epiblast

Naïve pluripotency is defined by the unrestricted developmental potential to give rise to all somatic lineages and the germline. In mouse, preimplantation epiblast cells isolated from the late ICM readily contribute to chimaeras when injected into a host blastocyst (Gardner and Rossant, 1979). Mouse ESCs can be captured from individual epiblast cells in naïve culture conditions and resemble the preimplantation epiblast both transcriptionally and functionally (Boroviak et al., 2014; Brook and Gardner, 1997). They efficiently contribute to chimaeras (Alexandrova et al., 2015; Ying et al., 2008), and ESCs that have downregulated the naïve marker *Zfp42* (*Rex1*) are predominantly eliminated from host embryos (Alexandrova et al., 2015). Thus, preimplantation epiblast identity is an integral feature of chimaera-competent ESCs.

**Fig. 3.**
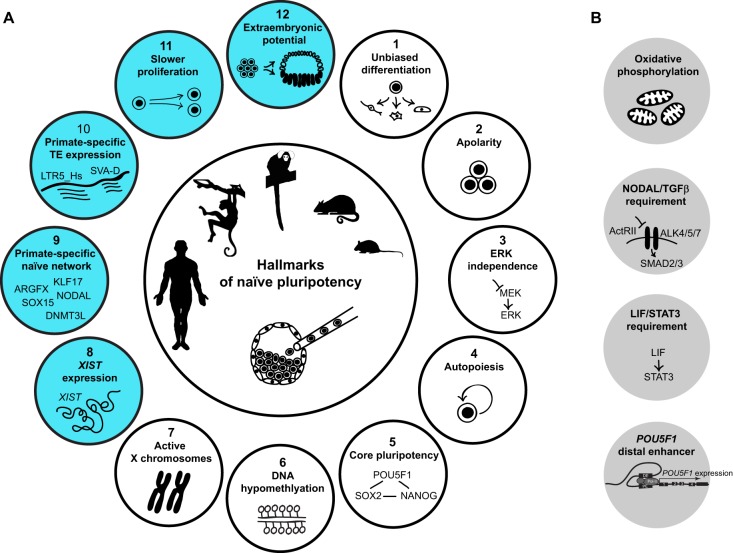
**Hallmarks of naïve pluripotency in primates.** (A) White circles symbolise hallmarks of naïve pluripotency in both rodents and primates, turquoise circles are specific to primates. (B) Grey circles encompass known features of the rodent preimplantation epiblast, which have not yet been analysed in primate embryos. TE, transposable element.

We propose that this imperative equally applies in primates. Therefore, the naïve state exists *a priori* in the preimplantation epiblast of the primate blastocyst. It represents a unique state of reset epigenome combined with a transcription factor configuration capable of delivering unbiased developmental plasticity. The naïve transcriptional circuitry has established control over genes required for cellular growth, organelle proliferation and lipid synthesis, abrogating the need for cleavage. It generates the first autopoietic cells of the embryo, equipped to establish secure and unconstrained nutrition by attachment to the uterus and to initiate the next steps of embryogenesis. Moreover, the proposition of a naïve state residing within the primate embryo does not entail that primate naïve pluripotency simply replicates the rodent paradigm. Primate naïve ESCs are expected to share more characteristics with rodent ESCs than with rodent EpiSCs, but in addition differences between naïve ESCs in rodents and primates are anticipated and even obligate. The next section of this article focuses on these conserved and distinctive features as we condense our current knowledge of the primate preimplantation epiblast into 12 hallmarks of naïve pluripotency (Fig. 3A). The first seven hallmarks equally apply to rodents and primates (white in Fig. 3A); the remaining five are specific to human and non-human primates (turquoise in Fig. 3A).

## Hallmarks of naïve pluripotency in primates

### 1. Unbiased differentiation potential

A defining feature of naïve pluripotency is the uncompromised ability to differentiate into somatic tissues and the germline *in vitro* and *in vivo* (Fig. 3A). Mouse EpiSCs and primed human ESCs display heterogeneity in their developmental potential, resulting in lineage bias (Bernemann et al., 2011; Bock et al., 2011; Han et al., 2010; Osafune et al., 2008). This variability in differentiation competence has been attributed to different levels of endogenous Wnt/β-catenin signalling (Blauwkamp et al., 2012; Davidson et al., 2012; Kurek et al., 2015). EpiSCs resemble the ectoderm of late gastrula stage embryos (Kojima et al., 2014), where Wnt/β-catenin signalling is pivotal for setting up anterior-posterior axis formation (Huelsken et al., 2000; Liu et al., 1999). Consistent with the rodent model, primate cells in the postimplantation embryo display increasing transcriptional heterogeneity towards gastrulation (Nakamura et al., 2016). In the naïve pluripotent epiblast, the transcriptional circuitry shields the cells from premature differentiation prior to implantation and preserves their full developmental potential.

### 2. Apolarity

In the outer blastomeres of the morula, establishment of polarity is associated with differentiation into the trophoblast lineage. Absence of polarity directs cells towards the inside of the embryo (Anani et al., 2014), an essential requirement for the establishment of pluripotency *in vivo* (Boroviak and Nichols, 2014). At the blastocyst stage, hypoblast precursor cells differentiate into an epithelium, while the pluripotent compartment remains sandwiched between the basal surfaces of trophoblast and hypoblast. In both rodents and primates, the preimplantation epiblast is an apolar cluster of cells (Bedzhov and Zernicka-Goetz, 2014; Enders et al., 1986; Plusa et al., 2005). This changes rapidly upon implantation, when epiblast cells arrange themselves into a rosette-like structure, concentrate their organelles at the apical end of the cell and form extensive adherence junctions (Bedzhov and Zernicka-Goetz, 2014; Enders et al., 1986). Acquisition of polarity paves the way for amniotic cavity formation, an essential process in all amniotes. *In vitro*, the apolar morphology of the preimplantation epiblast is preserved in the characteristic dome shape of naïve ESC colonies (Ying et al., 2008). By contrast, mouse EpiSCs and conventional human ESCs form flat colonies with ultrastructural characteristics similar to the postimplantation epiblast epithelium, including the presence of tight junctions and apical microvilli (Brons et al., 2007; Krtolica et al., 2007; Sathananthan et al., 2002; Tesar et al., 2007). The survival and proliferation of cells in epithelial structures is tightly controlled, rendering primed cells vulnerable to apoptosis upon single-cell dissociation (Ohgushi et al., 2010; Watanabe et al., 2007). This presents a major obstacle for successful chimaera formation upon blastocyst injection, but may be reversed in human reset ESCs maintained with aPKC inhibition (Takashima et al., 2014). aPKC is a kinase known to be essential for the establishment of apical-basal polarity from worms to man (Izumi et al., 1998; Suzuki et al., 2001). It is tempting to speculate that blocking the acquisition of epithelial polarity might interfere with differentiation towards a postimplantation epiblast state. Nevertheless, genetic studies will be required to ascertain the role of apical polarity proteins in the transition towards primed pluripotency.

### 3. ERK independence

Primed cultures require active FGF signalling. During blastocyst development, there is accumulating evidence that FGF/ERK inhibition promotes epiblast formation across species. In mouse, the FGF/ERK cascade is the predominant driver of hypoblast specification (Nichols et al., 2009; Yamanaka et al., 2010), whereas primates rely on additional signalling pathways (Boroviak et al., 2015; Kuijk et al., 2012; Roode et al., 2012). However, robust expression of NANOG in the absence of FGF/ERK signalling is reported in mouse, rat, bovine, marmoset and human blastocysts (Boroviak et al., 2015; Kuijk et al., 2012; Nichols et al., 2009; Roode et al., 2012). A recent study demonstrates similar findings in the zebra finch blastoderm (Mak et al., 2015), suggesting high conservation of the inverse correlation between naïve pluripotency and FGF/ERK signalling among amniotes.

### 4. Autopoiesis

The totipotent zygote undergoes cleavage divisions in the absence of cellular growth or increase in embryo mass. Cleavage occurs until the mid-blastocyst stage, when epiblast and hypoblast are specified (Aiken et al., 2004). The birth of naïve pluripotency in the epiblast is tightly linked to the establishment of autopoiesis (from the Greek meaning ʻself-creating'). In biology, autopoiesis refers to the ability of a cell to fully reproduce and maintain itself, that is, to ʻself-produce' all the same organelles, membranes and cytosolic components of which it is composed. This differs from the concept of self-renewal, which relates to the renewal of developmental potential and not necessarily the full self-renewal of cellular components per se. In mouse, embryonic cells gain autopoiesis just before implantation, when a safe and continuous nutrient supply is within reach (Aiken et al., 2004; Boroviak and Nichols, 2014). Cleavage stage and early ICM cells are unable to replenish their cytosolic compartment before cell division, precluding them from continuous and stable self-renewal (Boroviak et al., 2014; Boroviak and Nichols, 2014). In the primate embryo, precise measurements of nucleocytoplasmic ratios throughout preimplantation development are not yet available. However, measurements of cell sizes from histological sections (The Virtual Human Embryo, www.ehd.org/virtual-human-embryo/) suggest a decrease from morula and early ICM to the late blastocyst stage and constant or larger sizes at early postimplantation stages. Thus, it seems plausible that human and non-human primate embryos equally acquire autopoiesis around implantation. We suggest that autopoiesis is a hallmark of naïve pluripotency, distinguishing it from totipotent cleavage stages.

### 5. Core pluripotency

Pluripotency is conferred by a unique array of transcription factors. At the core of this network are *POU5F1*, *SOX2* and *NANOG*, which are evolutionarily conserved in mammals and several vertebrates (Dixon et al., 2010; Lavial et al., 2007; Tapia et al., 2012; Theunissen et al., 2011) and robustly expressed in both rodent and primate preimplantation epiblasts (Blakeley et al., 2015; Boroviak et al., 2015; Petropoulos et al., 2016). Interestingly, the core circuitry is shared between naïve and primed pluripotent cells, suggesting a context-dependent role in transcriptional regulation (Boiani and Scholer, 2005; Buecker et al., 2014). In mouse epiblast and ESCs, the framework is provided by naïve pluripotency factors such as *Klf2*, *Klf4*, *Klf5*, *Esrrb*, *Tfcp2l1*, *Tbx3* and *Zfp42*. This naïve circuitry is specifically expressed in pre- but not postimplantation development (Boroviak et al., 2014). Upon implantation in rodents, the wider pluripotency network is replaced with a different suite of transcription factors, including *Otx2*, *Pou3f1* (*Oct6*), *Sox3*, *Tead2* and *Bex1* (Acampora et al., 2013; Boroviak et al., 2015) to prepare the epiblast for gastrulation. *Nanog* is downregulated at early postimplantation stages in mouse. It has been proposed that during this formative phase, in the absence of both naïve factors and lineage specifiers, cells become receptive to differentiation stimuli (Kalkan and Smith, 2014; Smith, 2017). Subsequently, *Nanog* becomes re-expressed in the mouse posterior epiblast; localised expression of Wnt, Nodal and Bmp initiate primitive streak formation and establishment of the primary germ layers commences. Recent transcriptome profiling of non-human primate postimplantation stages revealed uninterrupted expression of *POU5F1*, *SOX2* and *NANOG* until gastrulation (Nakamura et al., 2016). This lends support to the crucial role of the core pluripotency network across developmental states.

### 6. DNA hypomethylation

The naïve character of the epiblast extends to epigenetic marks. DNA methylation carries important regulatory information and undergoes global resetting during germ cell and preimplantation development (Seisenberger et al., 2013a,b). In mouse and human, the preimplantation epiblast has a distinctive epigenetic signature consisting of genome-wide DNA hypomethylation with only the imprinted regions spared (Guo et al., 2014; Smallwood et al., 2011; Smith et al., 2014, 2012). This epigenetic status is preserved in mouse ESCs cultured in 2i/LIF, but not in serum-based conditions (Ficz et al., 2013; Habibi et al., 2013; Leitch et al., 2013). Conventional human ESCs exhibit high DNA methylation levels comparable to those of mouse EpiSCs, ESCs cultured in serum/LIF, or human somatic cells (Pastor et al., 2016). Resetting human ESCs with either 5i/L/FA (Theunissen et al., 2014) or t2iL+Gö (Takashima et al., 2014) induces hypomethylation at levels similar to the human ICM, but at the expense of DNA methylation of primary imprints (Pastor et al., 2016; Theunissen et al., 2016). This is problematic, since erroneous imprinting is implicated in a variety of human diseases and syndromes (Butler, 2009). Also, prolonged culture of human ESCs in 5i/L/FA leads to karyotypic abnormalities (Pastor et al., 2016; Theunissen et al., 2014), but whether loss of DNA methylation is the underlying cause remains to be elucidated.

### 7. Active X chromosomes

Most mammals exhibit random X-chromosome inactivation (XCI) in females to compensate X-linked gene expression (Escamilla-Del-Arenal et al., 2011; van den Berg et al., 2011). In mouse, the paternal X chromosome is silenced at the 4-cell stage (Huynh and Lee, 2003) and remains inactive in extraembryonic tissues. However, the paternal X chromosome is reactivated in the epiblast (Mak et al., 2004; Okamoto et al., 2004), tightly linked to the establishment of naïve pluripotency (Silva et al., 2009). In human blastocysts, transcription occurs from both X chromosomes in the preimplantation epiblast, as in mouse (Petropoulos et al., 2016). XCI ensues upon implantation and is associated with the establishment of primed pluripotency (Kobayashi et al., 2016). Thus, the presence of dually active X chromosomes is a hallmark of naïve pluripotency.

### 8. *XIST* expression

In mouse, XCI is mediated by the cis-acting, non-coding RNA *Xist*, which is downregulated in the epiblast. Re-expression occurs in the early postimplantation epiblast from either the maternal or paternal X chromosome to induce random XCI. By contrast, rabbit embryos initially upregulate *Xist* on both X chromosomes and, via an intermediate phase of biallelic XCI, induce random, monoallelic XCI at the late blastocyst stage before gastrulation (Okamoto et al., 2011). Consequently, biallelic X chromosome expression is not a hallmark of naïve pluripotency in the rabbit embryo.

Human embryos also lack paternal imprints for *XIST* expression, similar to rabbits, resulting in random XCI in both embryonic and extraembryonic lineages. However, despite biallelic *XIST* expression in more than half of the cells examined, both X chromosomes remain transcriptionally active in human blastocysts (Okamoto et al., 2011). Random XCI presumably occurs upon implantation, similar to in mice. Recent single-cell transcriptome data of human ICM cells confirm *XIST* expression at the blastocyst stage (Blakeley et al., 2015; Petropoulos et al., 2016; Yan et al., 2013). However, in contrast to mouse, dosage compensation occurs gradually in all three lineages of the human blastocyst, with both X chromosomes being actively transcribed throughout this process (Petropoulos et al., 2016). The mechanisms of dosage compensation in the presence of biallelic *XIST* expression remain unknown. Nevertheless, the embryo transcriptome data show that female human naïve pluripotent cells are expected to express *XIST* with both X chromosomes being active. This has been demonstrated recently for 5i/L/FA and t2iL+Gö reset cells (Sahakyan et al., 2016) and is in contrast to female naïve pluripotent cells in rodents, which do not express *XIST*.

### 9. Primate-specific naïve network

Naïve pluripotency factors are exclusive to preimplantation stages and sharply downregulated upon implantation and epiblast epithelialisation. Therefore, their identification relies on transcriptional analysis of both pre- and postimplantation samples. Naïve pluripotency factors in mouse include *Klf2*, *Klf4*, *Klf5*, *Stat3*, *Nr0b1*, *Esrrb*, *Tfcp2l1*, *Tbx3* and *Zfp42* (Boroviak et al., 2014, 2015; Peng et al., 2016; Scialdone et al., 2016). A subset of naïve factors, including *Stat3* (Yang et al., 2010), *Nr5a2* (Guo and Smith, 2010), *Klf2* (Hall et al., 2009), *Esrrb* (Festuccia et al., 2012), *Klf4* (Guo et al., 2009) and *Tfcp2l1* (Martello et al., 2013) can single-handedly drive naïve conversion from EpiSCs, and combinations of *NANOG* plus *KLF2* or *KLF4* have been used to reset human ESCs (Takashima et al., 2014; Theunissen et al., 2014).

The advent of single-cell profiling has allowed detailed molecular mapping of primate preimplantation development, and RNA sequencing (RNA-seq) datasets have become available in marmoset (Boroviak et al., 2015) and human (Blakeley et al., 2015; Petropoulos et al., 2016; Xue et al., 2013; Yan et al., 2013) showing that the majority of pluripotency-associated genes, including *POU5F1*, *SOX2*, *NANOG*, *SALL4*, *KLF4*, *TFCP2L1* and *TDGF1* are expressed in the primate epiblast. TFCP2L1, KLF4 and NANOG proteins colocalise in a subset of ICM cells in marmoset (Boroviak et al., 2015) and human (Takashima et al., 2014) blastocysts, suggesting partial conservation of the naïve circuitry. However, absence of *KLF2*, *ESRRB*, *NR0B1*, *FBXO15* and *BMP4*, and increased levels of *GDF3*, *NODAL*, *LEFTY1*, *KLF17* and *ARGFX*, demonstrate extensive primate-specific adaptation of the naïve pluripotency network (Blakeley et al., 2015; Boroviak et al., 2015; Petropoulos et al., 2016). Postimplantation stages in human are impossible to obtain for ethical reasons, but a recent report in cynomologus monkey provided a transcriptional blueprint from ICM to the late gastrula (Nakamura et al., 2016). Naïve markers expressed in the preimplantation epiblast but not in postimplantation stages included *TFCP2L1*, *KLF5*, *KLF17*, *NODAL* and *SOX15* (Nakamura et al., 2016). *KLF4* and *DNMT3L* were drastically downregulated upon implantation, but still expressed in the early postimplantation epiblast. The generation of chimaera-competent primate ESCs will rely on the complete re-establishment of the naïve circuitry that is operative in the preimplantation epiblast, free from expression of the mouse-specific *KLF2*, *ESRRB* and *NR0B1*.

### 10. Primate-specific transposable element (TE) expression

Global resetting of the epigenome during early development impacts on the expression of TEs, which make up half of the mammalian genome. Liberation from repressive DNA methylation in early developmental stages results in highly stage-specific TE expression (Göke et al., 2015). This transposcriptome has been proposed as an alternative measure to assess the correspondence between cultured pluripotent stem cells and the embryo (Theunissen et al., 2016). Human 5i/L/FA (Theunissen et al., 2014) and t2iL+Gö (Takashima et al., 2014) reset cells resemble human morula and blastocyst stages, respectively, showing elevated expression of the SINE-VNTR-*Alu* D subgroup (SVA-D) and LTR5_Hs (Theunissen et al., 2016). The close correlation to results from gene-based methods supports the overall conclusion of this new TE signature-based approach. However, while the transposcriptome may provide a more sensitive measure of the cell state in terms of transcript number, the functional relevance of similarities and divergences remains to be explored.

### 11. Slower proliferation

An important divergence between rodents and primates is the rate of proliferation. The mouse late blastocyst consists of ∼150 cells at embryonic day (E) 4.5 (Plusa et al., 2008), reflecting a cell cycle length of ∼15 h. Human embryos reach this stage after 7 days, having generated ∼250 cells (Niakan and Eggan, 2013). Thus, human embryonic cells have an increase in cell cycle length of at least 6 h, from 15 h to 21 h. Mouse ESCs exhibit comparable generation times (14-16 h) to their embryonic counterpart (Jovic et al., 2013), largely as a result of elevated and cell cycle-independent cyclin-dependent kinase 2 (Cdk2) expression (Stead et al., 2002). Cdk2 promotes the G1–S-phase transition by initiating DNA replication. By contrast, human (Blakeley et al., 2015; Yan et al., 2013) and marmoset (Boroviak et al., 2015) ICM cells lack constitutive *CDK2* expression, but show higher levels of *WEE1*, a key cell cycle inhibitor. This demonstrates major differences in the cell cycle machinery between rodents and primates. Thus, authentic primate ESCs are not expected to typify their rodent counterparts with regard to proliferation rates.

### 12. Extraembryonic potential

The divergence of rodent and primate postimplantation development transforms the concept and prospects of naïve pluripotency in primates. In contrast to mouse epiblasts, primates segregate an additional lineage before gastrulation, whereby the proximal epiblast differentiates into amniotic epithelium (Enders and Lopata, 1999; Enders et al., 1986). We therefore hypothesise that authentic human naïve pluripotent cultures should have an expanded capacity to produce both postimplantation epiblast and amniotic epithelial cells. This means that naïve primate ESCs should be able to differentiate into either cell type within a short time window. However, currently there are two key pieces of information missing: (1) the signalling pathways that control this lineage decision; and (2) the transcriptional and epigenetic signature of amniotic epithelial cells. A clear understanding of the developmental cues that determine amnion differentiation will be required to specify this extraembryonic lineage efficiently from naïve primate ESCs *in vitro*. Moreover, this experiment demands a detailed knowledge of the molecular signature of amniotic epithelial cells *in vivo* for meaningful endpoint analysis. Future studies of non-human primate postimplantation development including samples of amniotic epithelial cells and tracking of spatial identity within the embryo might be able to tackle these questions.

## The 12 hallmarks: a testable framework for human naïve ESCs

We propose that the 12 hallmarks of naïve pluripotency outlined above can constitute a powerful system to assess human naïve pluripotency *in vitro*. Primate cells in a naïve state are expected to tolerate long-term MEK inhibition via PD0325901 (hallmark 3) and to grow more slowly than mouse ESCs (hallmark 11) in apolar, dome-shaped colonies (hallmark 2). Absence of epithelial character can be further examined by antibody staining for apical polarity and tight junction proteins. The autopoietic nature of the cells allows stable long-term propagation (hallmark 4), distinguishing them from totipotent cells, which cannot be propagated indefinitely. Hypomethylation can be evaluated by bisulphite sequencing (hallmark 6). Genome-wide transcriptional profiling by RNA-seq permits testing for core pluripotency (hallmark 5), *XIST* expression (hallmark 8), the primate-specific naïve network (hallmark 9) and TE expression (hallmark 10). Read lengths of more than 100 bp are favourable to facilitate mapping of highly repetitive TEs. Moreover, exploring the wider naïve transcriptional circuitry and the TE signature are powerful ways to discriminate between primate epiblast identity and artificial mouse ESC-like states. Absence or low-level expression of mouse-specific pluripotency factors, including *KLF2*, *ESRRB*, *NR0B1* and *FBXO15*, are important indicators for successful resetting towards an authentic human epiblast state. High-quality RNA-seq datasets may also be used to detect SNPs and assess biallelic expression from the X chromosome (hallmark 7). Alternatively, the X-chromosome activation status can be determined by fluorescence *in situ* hybridisation (hallmark 7).

In addition to descriptive analysis, it is pivotal to test functionally unbiased differentiation potential (hallmark 1) and extraembryonic capacity for amnion formation (hallmark 12). Human germline chimaera contribution assays are prohibited on ethical grounds. However, unbiased differentiation can be gauged *in vitro* and by teratoma formation *in vivo*. From a developmental point of view, naïve ESCs are expected to differentiate into somatic lineages via successive formative and primed pluripotent states (Smith, 2017). This needs to be considered when applying stepwise protocols for directed differentiation. Epigenetic resetting to the naïve state may eradicate some of the lineage bias observed in conventional human ESCs. These experiments demand careful quantification of various differentiation assays and would only become meaningful after comparing multiple independent lines. Moreover, it is difficult to discern genetic diversity from epigenetic lineage bias. In practice, quantitative differentiation might not be suitable for routine assessment of naïve pluripotency.

The specific ability of the primate peri-implantation epiblast to give rise to nascent amnion (hallmark 12) might provide a more explicit functional assay to discriminate between naïve and primed states. Conventional human ESCs correspond to the pregastrula embryonic disc (Nakamura et al., 2016), 7 days after amnion segregation. Naïve pluripotency is established in the epiblast just before this decision point. Thus, naïve human ESCs should be competent to replicate amnion segregation and amniotic cavity formation of postimplantation stages. Stimuli from the extracellular matrix and/or adjacent extraembryonic tissues might be essential for this transition. The recent reports on amniotic cavity formation of human embryos cultured to postimplantation stages *in vitro* (Deglincerti et al., 2016; Shahbazi et al., 2016) lend support to the feasibility of this undertaking. An *in vitro* system to obtain and study human embryonic and extraembryonic lineages from cultured cells would be highly desirable to unravel the continuum of pluripotent states in the primate embryo.

## Unresolved issues in primate development

Several features of naïve pluripotency remain uncertain in primates (Fig. 3B). Mouse ESCs are bivalent in their energy production, using both oxidative phosphorylation and glycolysis, whereas EpiSCs shift their metabolism to high glycolysis, phenotypically akin to rapidly proliferating cancer cells (Zhou et al., 2012). A number of recent studies have characterised metabolic dynamics in different pluripotent states *in vitro* (reviewed by Teslaa and Teitell, 2015); however, whether this paradigm applies to bona fide primate embryonic development remains unclear. While quantitative measurements of metabolites or oxygen consumption rates are difficult to obtain *in vivo*, results from *in vitro* derived cells might not reflect the situation in the embryo. For example, it has been suggested that nicotinamide N-methyltransferase (NNMT) regulates a metabolic switch between human primed and putative naïve ESCs cultured in 2i/FGF (Sperber et al., 2015). However, NNMT is not expressed in the human preimplantation epiblast (Blakeley et al., 2015; Petropoulos et al., 2016; Yan et al., 2013). Another contentious subject is NODAL/TGFβ signalling in the primate blastocyst. Human embryos cultured in the presence of the NODAL/TGFβ inhibitor SB431542 are reported to increase the number of NANOG-positive ICM cells (Van der Jeught et al., 2014). However, similar experiments using higher concentrations showed a dramatic reduction of NANOG expression (Blakeley et al., 2015). In marmoset, NODAL/TGFβ inhibition with A83-01 did not modulate NANOG expression (Boroviak et al., 2015). The question of whether NODAL/TGFβ signalling is functionally required for primate naïve pluripotency is interesting and deserves further attention. Equally unclear is the role of LIF/STAT3 signalling or whether *POU5F1* expression in the embryo primarily relies on its distal enhancer. So far, specific *POU5F1* distal enhancer operation has not been demonstrated in the primate epiblast. Further refinements of ChIP-seq and advanced chromosome configuration capture approaches for single-cell analysis will help to address some of these questions.

## Future perspectives of naïve pluripotency in primates

The capture of authentic developmental states forms an integral part of both basic and applied research. Naïve ESCs provide a tool to functionally assess the factors that control *in vivo* development. This is of particular importance in primates, where embryonic material is precious and scarce. Second, robust differentiation of pluripotent cells relies on a precise spatiotemporal sequence of specification events. A defined developmental starting point is essential to mimic embryonic patterning *in vitro*. Preimplantation epiblast identity delivers an exact developmental stage with well-defined characteristics (Fig. 3A), in addition to favourable cell biology features such as apolarity for efficient single-cell cloning. Despite the remarkable success of designer nucleases (Liu et al., 2014; Sato et al., 2016) and Cas9/RNA-mediated gene targeting (Niu et al., 2014) in non-human primate zygotes, it is technically and economically challenging to obtain sufficient numbers of primate embryos for knock-in strategies. Currently, this limits gene-editing approaches to simple gene disruption.

Chimaera-competent ESCs in non-human primates might open up avenues for sophisticated genetic engineering to create versatile models for basic and preclinical research. This is important in areas where rodent models are insufficient, including infectious diseases, neurodegenerative disorders, aging and reproductive medicine (Carrion and Patterson, 2012; Mansfield, 2003; Okano et al., 2012; Shedlock et al., 2009). Another emerging application for naïve ESCs in biomedical research is organ farming. Rat ESCs are capable of filling the developmental niche of mouse *Pdx1* (pancreatogenesis-disabled) null host embryos (Kobayashi et al., 2010), a procedure referred to as interspecies chimaeric complementation (reviewed by Wu and Izpisua Belmonte, 2015). This concept might be exploited to grow human organs in pigs for xenotransplantation. The recent generation of apancreatic pigs provides another key step towards clinical application (Matsunari et al., 2013). However, the lack of chimaera-competent primate ESCs currently presents a bottleneck for the generation of primate organs in farm animals. In addition, there are ethical concerns with regard to unwanted tissue contribution of human cells to the pig central nervous system or gametes. The use of naïve non-human primate ESCs in interspecies chimaeric complementation will be pivotal to resolve these issues and turn the xenomedical vision into reality.
